# Low pressure hyperbaric oxygen therapy and SPECT brain imaging in the treatment of blast-induced chronic traumatic brain injury (post-concussion syndrome) and post traumatic stress disorder: a case report

**DOI:** 10.1186/1757-1626-0002-0000006538

**Published:** 2009-06-09

**Authors:** Paul G Harch, Edward F Fogarty, Paul K Staab, Keith Van Meter

**Affiliations:** 1 Section of Emergency Medicine, Department of Medicine, Louisiana State University Health Sciences Center, 2021 Perdido St, Room W535, New Orleans, Louisiana, 70112, USA; 2 Department of Radiology, University of North Dakota School of Medicine and Health Sciences, Post Office Box 1975, 515 ½ East Broadway Avenue, Suite 106, Bismarck, North Dakota, 58502, USA

## Abstract

A 25-year-old male military veteran presented with diagnoses of post concussion syndrome and post traumatic stress disorder three years after loss of consciousness from an explosion in combat. The patient underwent single photon emission computed tomography brain blood flow imaging before and after a block of thirty-nine 1.5 atmospheres absolute hyperbaric oxygen treatments. The patient experienced a permanent marked improvement in his post-concussive symptoms, physical exam findings, and brain blood flow. In addition, he experienced a complete resolution of post-traumatic stress disorder symptoms. After treatment he became and has remained employed for eight consecutive months. This case suggests a novel treatment for the combined diagnoses of blast-induced post-concussion syndrome and post-traumatic stress disorder.

## Introduction

By January, 2008 it was estimated that as many as 300,000 servicemen and women from the current Iraq and Afghanistan Wars have PTSD or major depression, 320,000 have experienced a TBI, and 82,000 have all three diagnoses [1]. Treatment is available for PTSD and depression, but there is no proven therapy for the dual diagnoses of PTSD and the residual effects of TBI, the PCS [2].

HBOT is the use of greater than atmospheric pressure oxygen in an enclosed chamber to treat basic disease processes [3]. HBOT has been traditionally applied to certain emergent conditions and chronic wound conditions, but not to blast-induced TBI/PCS or PTSD. This case report is the first application of the authors' low pressure HBOT protocol for chronic brain injury to blast-induced TBI/PCS and PTSD. An early version of this protocol was recently reported in an animal model of chronic TBI that duplicated the human experience [4].

## Case presentation

A 25-year-old retired Caucasian male U.S. Marine presented with headaches, tinnitus, and sleep disturbance. Three years before evaluation the patient sustained LOC (a few minutes) from an IED explosion with anterograde memory loss and confusion (one hour), and persistent right ear tinnitus, headaches, imbalance, and sleep disturbance. He developed PTSD symptoms within 3 months and experienced six more explosions with near LOC within 15 months. After medical evaluation diagnoses were TBI/PCS, PTSD, depression, hearing loss, and tinnitus.

**Prioritized Symptom List**: 1) Constant headaches with intermittent confusion, irritability, tunnel vision, and dizziness, 2) Bilateral tinnitus, 3) Sleep disruption, 4) Left eye blurred vision, 5) Irritability, 6) Depression, social withdrawal; **Additional Symptoms**: 7) Fatigue, 8) Decreased hearing, 9) Imbalance, 10) Cognitive problems-memory, attention, decreased speed of thinking, 11) Back pain, 12) Bilateral knee pain, 13) PTSD symptoms: intrusive thoughts, combat thoughts, nightmares, tachycardia.

Med-Surg, Medications: None. **FH, ROS, and PHIS**: non-contributory or negative. **PSH**: Engaged, no children, lives with parents, 3 years college education, no tobacco or drugs, one to two beers/week. **Neuro PEx Abnormalities**: Slight deviation of right eye laterally, bilateral: decreased hearing to softly rubbing fingers at one foot, noxious response to 512 Hz tuning fork, decreased finger tapping speed, unstable: rotation exam, tandem gait, and Romberg. **Treatment and testing:** MRI brain-normal. SPECT brain imaging pre-HBOT and 72 h after the 39^th^ HBOT. The patient underwent 39 HBOT's in 26 calendar days at 1.5 ATA/60 minutes total dive time, twice/day, five days/week in a monoplace chamber with 100% oxygen.

**Figure 1:**
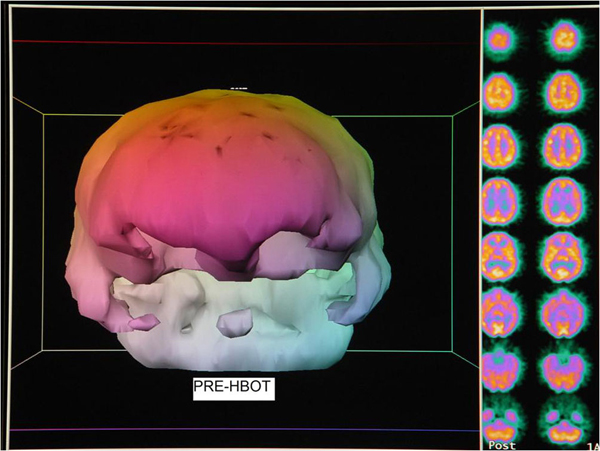
**Pre-HBOT SPECT brain scan three dimensional surface reconstruction and processed transverse images**. Note bilateral orbital frontal and temporal lobe defects and diffuse heterogeneous pattern of blood flow.

**Figure 2:**
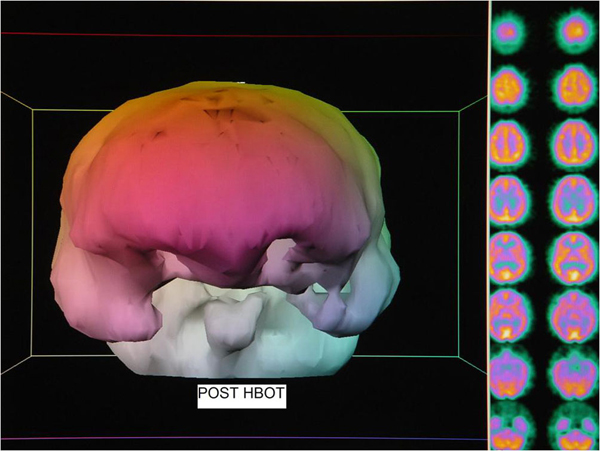
**Post-HBOT SPECT brain scan three dimensional surface reconstruction and processed transverse images**. Note relative improvement in brain blood flow to bilateral focal frontal and temporal defects and overall normalization of blood flow to a more homogeneous pattern.

**Outcome:** Headache permanently gone after the 1^st^ HBOT. After 12 HBOT's symptoms 3, 6, and 7 improved. At 25^th^ HBOT absence of PTSD symptoms. Re-evaluation after 37 HBOT's: 1) 4/6 primary problems improved (#'s 1, 3, 5, 6), 2/6 no change, 2) 4/7 additional symptoms improved (7, 9, 10, 13), 3/7 no change, 3) 6/6 abnormal exam findings retested improved, 1 finding not retested (right eye deviation). SPECT: heterogeneous with bilateral frontal and temporal defects-all improved post HBOT. (See Additional file 1, Figures 1 and 2. (Figure 1): Pre-HBOT SPECT brain scan three dimensional surface reconstruction and processed transverse images. Pre-HBOT scan was rendered in three dimensional surface reconstruction format by PJT based on the method developed and taught by Picker International using Picker software. In this method brain blood flow is computer indexed to frontal lobe blood flow. A frontal lobe surface defect was identified on a selected transverse slice. Processed/filtered transverse slices were then featured with a 100% window such that all pixels render a white image. Counts were slowly subtracted by decreasing the window threshold until the defect was visible as a full thickness black defect in the contour of the cortex. As the defect emerged and was registered in proper anatomic proportion to the rest of frontal cortical blood flow the numerical window level was taken as the determination threshold. Three separate determinations were made for each scan and the final threshold taken as an average of the three determinations. The technologist was blind to the final image reconstruction due to software restrictions that only allow threshold determination. The surface reconstruction image at this threshold is featured in the image above. Color is aesthetic. Note bilateral orbital frontal and temporal lobe defects, areas typically injured in traumatic brain injury, consistent with processed transverse images in the right hand columns. Processed images also show an abnormal diffuse heterogeneous pattern of blood flow. Description of processing is in (SD1). (Figure 2): Post-HBOT SPECT brain scan three dimensional surface reconstruction and processed transverse images. Three dimensional surface image was prepared in identical fashion to the image in Figure 1. Note relative improvement in brain blood flow to bilateral focal frontal and temporal defects, consistent with processed transverse images in the right hand columns. Transverse slices also show normalization of the blood flow to a more homogeneous pattern.

## Discussion

The present case is the first application of the author's HBOT protocol to blast-induced TBI/PCS and PTSD. The patient's symptomatic, physical exam, and SPECT improvements are similar to ours [3,5,6,9] and others' [7,8] previous cases/case series of non-blast TBI suggesting common pathophysiology. The unexpected result was the complete resolution of PTSD. With the overlap of symptoms, pathophysiology, and anatomy in TBI/PCS and PTSD [10] HBOT is likely impacting common shared targets in this case.

## Conclusion

Thirty-nine low pressure HBOT's caused a reduction in symptoms and signs of chronic mild-moderate blast-induced TBI/PCS and PTSD. The resolution of symptoms and signs of TBI/PCS and PTSD were reflected in global and focal improvements in brain blood flow imaging, suggesting a novel treatment for these combined diagnoses.

## Patient's perspective

Patient has declined to submit his perspective due to privacy concerns.

## List of abbreviations

ATA: Atmospheres absolute; ECD: Ethyl cysteinate dimer; FH: Family history; HBOT: Hyperbaric oxygen therapy; HPI: History of present illness; IED: Improvised explosive device; LOC: Loss of consciousness; MRI: Magnetic resonance imaging; PCS: Post-concussion syndrome; PEx: Physical exam; PHIS: Prior head injury history; PMH: Past medical history; PSH: Personal and Social history; PTSD: post-traumatic stress disorder; ROS: Review of systems; SPECT: Single photon emission computed tomography; TBI: Traumatic brain injury.

## Consent

Written informed consent was obtained from the patient for publication of this case report and accompanying images. A copy of the written consent is available for review by the Editor-in-Chief of this journal. In addition, this case was approved by the LSU School of Medicine's Institutional Review Board as a case report.

## Competing interests

The authors declare competing interests. The primary author has a small corporation, Harch Hyperbarics, Inc. that does hyperbaric consulting. Author KVM has a corporation that leases hyperbaric oxygen chambers and a corporation that contracts to provide hyperbaric oxygen and woundcare services. None of the authors have personal or financial relationships with people or organizations that would influence the interpretation of data in this report.

## Authors' contributions

PGH evaluated the patient, ordered the treatment and imaging, and wrote the draft of the manuscript. EFF analyzed and presented the SPECT imaging and assisted in writing the manuscript. PKS assisted in the treatment of the patient and assisted in writing the manuscript. KVM assisted in development of the hyperbaric protocol and writing the manuscript. All authors read and approved the final manuscript.

## Supplementary Material

Additional file 1 **Side by side Pre and Post HBOT processed transverse SPECT brain blood flow images**. Pre-HBOT scan is on the left and post-HBOT on the right. Click on either image to initialize movie. Images were obtained on a Picker Prism 3000 triple-head gamma camera. Both scans were processed by technologist PJT: 25 mCi of ECD was prepared with the standard manufacturer's kit and injected in a peripheral vein in a low noise low light area while the patient was quiet and motionless. One hour after injection acquisition proceeded with a 360 degree rotation and 40 stops, 20 seconds/stop on a 128 x 128 matrix, using low energy high resolution fan beam collimators. Motion correction was used for minor movement. Raw data was processed by transverse reconstruction using 360 degree filtered back projection and a ramp filter, followed by a LoPass filter, order 2.2. Cutoff was taken at the intersection of the best fit LoPass filter and noise on the power spectrum graph. Per file attenuation correction and best fit ellipse were applied. Images were oblique reformatted with slice thickness at 4 mm (2 pixels), aligned, and off-center zoom applied (20 cm^2^ area). Images were presented in all 3 orthogonal planes. Transverse processed images were analyzed with Osirix Open-source software (version 3.3.2) and windowed at a level of 1000 with a window width of 2000. They were subsequently rendered in QuickTime movie format starting from vertex and proceeding through the base of the brain. Images are in standard SPECT format and orientation. Color map is red, yellow, green, blue, and violet from highest brain blood flow to lowest. Note the marked generalized increase in perfusion on the post-HBOT scan Click here for file

## References

[B1] Tanielian TJaycox LH Invisible Wounds of War: Psychological and Cognitive Injuries, Their Consequences, and Services to Assist Recovery 2008 Center for Military Health Policy Research, the Rand Corporation

[B2] King NS PTSD and traumatic brain injury: Folklore and fact? Brain Injury 2008 22 1 5 10.1080/02699050701829696 18183503

[B3] Harch PG Neubauer RA Jain KK The Textbook of Hyperbaric Medicine 1999 3 Hogrefe and Huber 318 349

[B4] Harch PG Kriedt C Van Meter KW Sutherland RJ Hyperbaric oxygen therapy improves spatial learning and memory in a rat model of chronic traumatic brain injury Brain Res 2007 1174 120 129 10.1016/j.brainres.2007.06.105 17869230

[B5] Harch PG Van Meter KW Neubauer RA Gottlieb SF Jain KK The Textbook of Hyperbaric Medicine 1996 2 Hogrefe and Huber 480 491

[B6] Harch PG Neubauer RA Jain KK Hyperbaric oxygen therapy in global cerebral ischemia/anoxia and coma The Textbook of Hyperbaric Medicine 2004 4 Hogrefe & Huber 223 262

[B7] Neubauer RA Gottlieb SF Pevsner NH Hyperbaric oxygen treatment of closed head injury South Med J 1994 87 933 936 8091261 10.1097/00007611-199409000-00015

[B8] Golden ZL Neubauer RA Golden CJ Improvement in cerebral metabol-ism in chronic brain injury after hyperbaric oxygen therapy Int J Neurosci 2002 112 119 131 10.1080/00207450212027 12325401

[B9] Harch PG Gottlieb SF Van Meter KW Staab P HMPAO SPECT brain imaging and low pressure HBOT in the diagnosis and treatment of chronic traumatic, ischemic, hypoxic and anoxic encephalopathies Undersea & Hyperbaric Medicine 1994 21 30

[B10] Kennedy JE Jaffee MS Leskin GA Posttraumatic stress disorder and posttraumatic stress disorder-like symptoms and mild traumatic brain injury J Rehab Res Devel 2007 44 895 920 10.1682/JRRD.2006.12.0166 18075948

